# Oral Morphine Solution as an Oral Rinse or Mouth Gargle for Mucositis Pain

**DOI:** 10.4103/0973-1075.63138

**Published:** 2010

**Authors:** G Saroja, P Saraswathi Devi, R Namrata

**Affiliations:** Department of Anesthesiology and Palliative Care Unit, Kidwai Memorial Institute of Oncology, Bangalore, Karnataka, India

Sir,

Oral mucositis is an inflammatory-like process of the oral mucosa due to radiotherapy and chemotherapy. This is characterized by atrophy of squamous epithelial tissue, vascular damage, and inflammatory infiltration concentrated at the basement region. This becomes evident of aching ulcerative abrasions of mouth and even throat. Pain and dysphagia (restriction of oral intake) due to oral mucositis may further debilitate the immunocompromised patients. Patients having acute oral mucositis will find it extremely tough to perform simple everyday mouth-related actions like chewing, biting, talking, swallowing, drinking, sipping, etc.

Treatment of mucositis is still limited to the reduction of severity of pain and discomfort due to mucositis. Several anesthetics, analgesics, and mucosal agents have been used.

The main challenges to the palliative care professionals posed by the patients with extensive and severe ulcerative lesions in the oropharynx are to maintain the nutrition and hydration, involve them in conversation, and expect them to communicate with the team. Keeping the above facts in mind and observing the severity of mucositis pain experienced by many patients led us to initiate the study using oral morphine solution as oral morphine rinse.

Ten patients with extensive oral mucositis admitted in palliative care ward were recruited for the study. Regular pain assessment was done by visual analog scale at the time of admission and everyday in the ward. Oral morphine solution used as rinse for the study is prepared according to Royal Marsden Hospital formula at the pharmacy in palliative care unit. The solution has strength of 5 mg morphine sulphate in 15 ml of diluent solution given every 2 h[1] and instructed to keep it in mouth for 5 min and then spit it out. The side-effects were monitored and reported to the team members and to the ward staff in the night. All 10 patients were only on oral rinse of oral morphine solution for 48 h but not on any other step III drugs by other routes. Seven patients were on NSAIDs and three patients were on antifungal, antibiotics before starting oral morphine rinse. These patients with severe mucositis have adapted to their comfortable way of taking oral feeds. Patients were advised to do the oral rinse in the same way or method.

All patients experienced good pain relief after 15 min of oral rinse and this lasted for 30–60 min. It was observed that the mouth opening had increased by 0.5–1.5 cm. Two patients complained of itching and burning sensation at the site of the lesion after 24 h; two patients had bleeding during rinsing; and one patient complained of giddiness.[1,2] Patients found initial difficulty to rinse because of the restricted movement of mouth opening due to trismus.

Oral morphine solution used as oral rinse is prepared according to the Royal Marsden Hospital formula using morphine sulphate powder. This solution is prepared at pharmacy in palliative care unit and dispensed according to the patients’ requirements.

Patients included in this study had come with the complaints of odynophagia, oral mucositis pain, and severe trismus. Patients were given 5 mg morphine sulphate in 15 ml of diluent solution[1] given every 2 h for rinse as 10 mg every 4 h is the starting dose of morphine for step II failures.

Patients were able to open their mouth by 0.5–1.5 cm; however the intensity of mucositis-related pain, though less, was persisting. Pain relief was achieved to the maximum of 1 h after oral rinse. As patients were not relieved of pain with oral rinse alone, they were administered with routine immediate release morphine solution as per WHO analgesic ladder along with oral rinse for effective continuous relief of pain.

A combination of oral morphine rinse every 2 h along with the intake of morphine solution every 4 h proved to be effective in relieving the pain of mucositis and improving the opening of their mouth.
